# Transcriptome analysis of axillary buds in low phosphorus stress and functional analysis of *TaWRKY74*s in wheat

**DOI:** 10.1186/s12870-023-04695-w

**Published:** 2024-01-02

**Authors:** Xue-zheng Li, Xiao-tong Zhang, Xiao-min Bie, Jing Zhang, Deng-ji Jiang, Heng Tang, Fang Wang

**Affiliations:** 1 National Key Laboratory of Wheat Breeding, Shandong Agricultural University, Taian, Shandong 271018 China; 2 College of Life Sciences, Shandong Agricultural University, Taian, Shandong 271018 China; 3 College of Agriculture, Shandong Agricultural University, Taian, Shandong 271018 China; 4 College of Plant Protection, South China Agricultural University, Guangzhou, 510000 China

**Keywords:** Wheat, Low phosphorus stress, *TaWRKY74*s, Functional analysis

## Abstract

**Background:**

Wheat is one of the main grain crops in the world, and the tiller number is a key factor affecting the yield of wheat. Phosphorus is an essential element for tiller development in wheat. However, due to decreasing phosphorus content in soil, there has been increasing use of phosphorus fertilizer, while imposing risk of soil and water pollution. Hence, it is important to identify low phosphorus tolerance genes and utilize them for stress resistance breeding in wheat.

**Results:**

We subjected the wheat variety Kenong 199 (KN199) to low phosphorus stress and observed a reduced tiller number. Using transcriptome analysis, we identified 1651 upregulated genes and 827 downregulated of genes after low phosphorus stress. The differentially expressed genes were found to be enriched in the enzyme activity regulation related to phosphorus, hormone signal transduction, and ion transmembrane transport. Furthermore, the transcription factor analysis revealed that *TaWRKY74*s were important for low phosphorus tolerance. *TaWRKY74*s have three alleles: *TaWRKY74*-A, *TaWRKY74*-B, and *TaWRKY74*-D, and they all belong to the WRKY family with conserved WRKYGQK motifs. These proteins were found to be located in the nucleus, and they were expressed in axillary meristem, shoot apical meristem(SAM), young leaves, leaf primordium, and spikelet primordium. The evolutionary tree showed that *TaWRKY74*s were closely related to *OsWRKY74*s in rice. Moreover, *TaWRKY74*s-RNAi transgenic plants displayed significantly fewer tillers compared to wild-type plants under normal conditions. Additionally, the tiller numebr of the RNAi transgenic plants was also significantly lower than that of the wild-type plants under low-phosphorus stress, and increased the decrease amplitude. This suggestd that *TaWRKY74*s are related to phosphorus response and can affect the tiller number of wheat.

**Conclusions:**

The results of this research showed that *TaWRKY74*s were key genes in wheat response to low phosphorus stress, which might regulate wheat tiller number through abscisic acid (ABA) and auxin signal transduction pathways. This research lays the foundation for further investigating the mechanism of *TaWRKY74*s in the low phosphorus environments and is significant for wheat stress resistance breeding.

**Supplementary Information:**

The online version contains supplementary material available at 10.1186/s12870-023-04695-w.

## Introduction

Wheat is one of the main food crops in the world. The yield per unit area is determined by the spike number per unit area, grain number per spike, and grain weight per spike [1]. Notably, these three factors not only interact with each other but also constrain each other. The tiller number determines the effective spike number to a certain extent, thus affecting the yield [2]. The axillary bud is the initial state of the tiller, its development in wheat directly affects tiller number. So, studying axillary buds is an effective approach to better understand tillering.

Phosphorus plays a vital role in both the morphological structure and growth of plants [3]. For example, phosphorus is involved in the regulation of enzyme activity, synthesis of nucleic acids and biological membrane, and regulation of energy metabolism [4]. Phosphorus was also reported to be important for the development of axillary buds; in a low phosphorus environment, both tillering and axillary buds are inhibited in rice [5]. The growth of axillary buds is controlled by hormones, and phosphorus is an important factor involved in the synthesis of hormones. For instance, phosphates can regulate the synthesis of strigolactone, which participates in the outgrowth of axillary buds in rice. Hence, phosphorus may regulate the development of axillary buds by affecting hormones [6].

At present, a lot of arable land globally is facing the problem of low phosphorus [7]. To increase crop yields, farmers often apply phosphorus-containing fertilizers during the cultivation process. However, a large amount of phosphorus fertilizer may cause soil salinization, deterioration of physical and chemical properties of soil, and flow of excess phosphorus into the water, leading to eutrophication [8]. Therefore, it is essential to identify low phosphorus tolerance genes and cultivate crops that carry these genes [9].

The WRKY protein family constitutes a plant-specific transcription factor (TF), and WRKY protein plays an important role in nutrient deficiency response [10]. The most prominent feature of the WRKY proteins is the presence of the WRKY domain, which consists of 60 amino acids with a highly conserved N-terminal amino acid sequence of WRKYGQK [11]. WRKY proteins are classified as I, II, and III according to the number of WRKY protein domains and the type of zinc finger motifs [12]. Type III WRKY TFs are only present in higher plants and are mostly associated with the responses to stress [13]. When *Arabidopsis* were subjected to low phosphorus stress, *AtWRKY75* was strongly induced in plants, whereas the inhibition of *AtWRKY75* expression led to increased sensitivity of mutant plants to inorganic phosphate (Pi) stress and decreased uptake of Pi [14]. *AtWRKY6* negatively regulated phosphorus transport by inhibiting the low-phosphorus regulatory gene *AtPHO1* expression [15]. *AtWRKY45* was involved in the response of *Arabidopsis* to Pi starvation by directly upregulating *PHT1*. During phosphorus deficiency, the expression of *AtWRKY45* was significantly induced, especially in roots [16]. A few WRKY TFs regulated low-phosphorus adaptation [17]. For example, the overexpression of rice *OsWRKY74* significantly enhanced the tolerance of transgenic plants to low phosphorus stress by activating phosphorus starvation-induced genes and regulating root structure, whereas OsWRKY74-silent strains were more sensitive to low phosphorus stress [18].

In this study, transcriptomic analyses of axillary buds from the wheat Kenong199(KN199) which was treated with low phosphorus stress were performed. The differential expression genes *TaWRKY74*s were selected. We cloned the genes to analyze their function and constructed *TaWRKY74*s-RNAi transgenic plants. The results showed that *TaWRKY74*s played an important role in response to low phosphorus stress and might regulate tiller number through abscisic acid (ABA) and auxin signal transduction pathways in wheat.

## Results

### Effects of different concentrations of phosphorus treatment on wheat

**Fig. 1:**
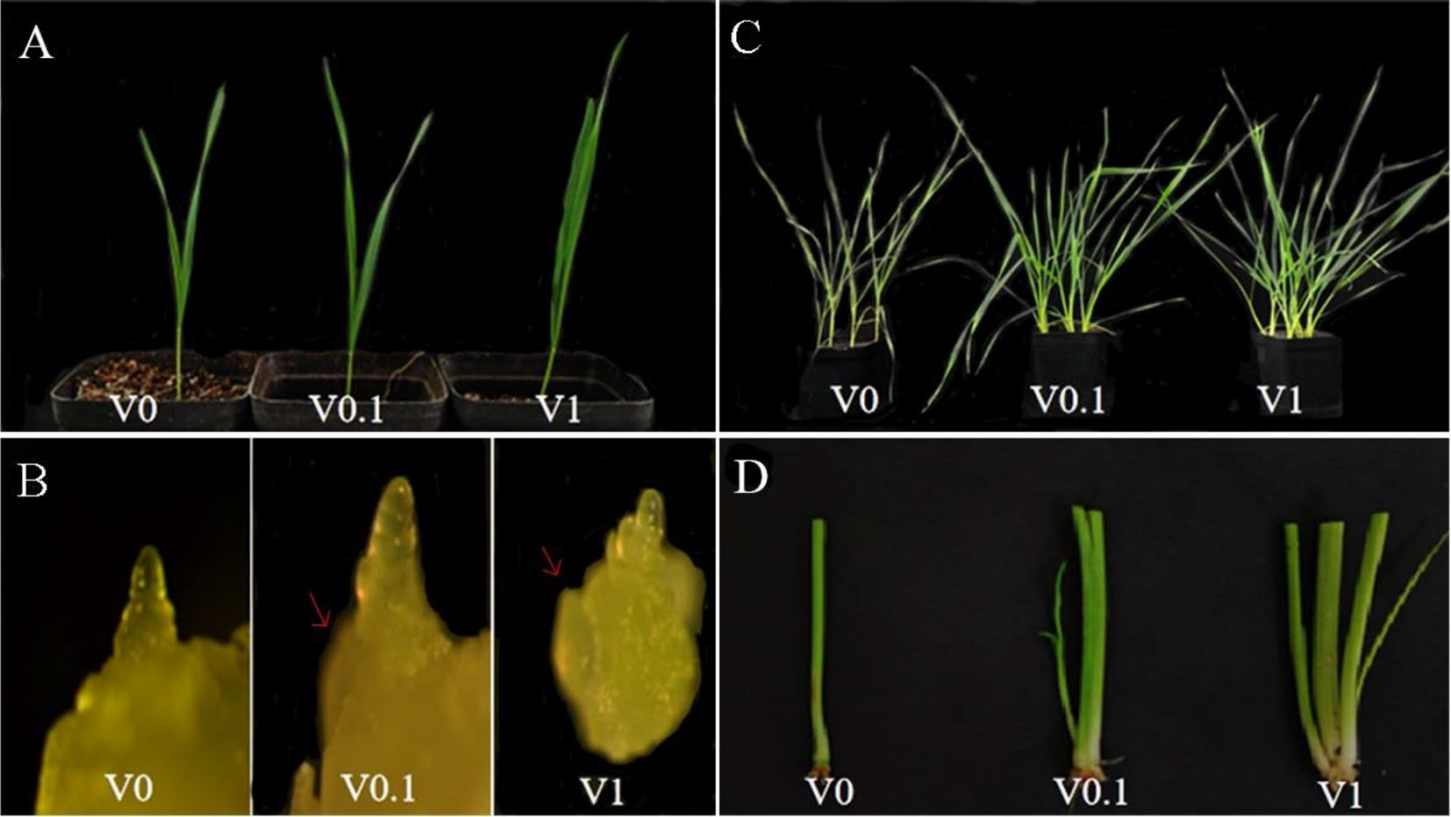
Phenotypic observation of wheat under V0, V0.1, and V1 treatment. (**A**) Phenotypic observation of KN199 after V0, V0.1, and V1 treatment on day 8 in the greenhouse. (**B**) Comparative observation of KN199 stem apical meristem and tiller nodes after V0, V0.1, and V1 treatment on day 8 in the greenhouse. (**C**) Phenotypic observation of KN199 after V0, V0.1, and V1 treatment on day 20 in the greenhouse. (**D**) Comparative observation of KN199 tiller after V0, V0.1, and V1 treatment on day 20 in the greenhouse

After transferred to the greenhouse, the wheat cultivar KN199 was grown in same conditions for the first 5 days. On day 6, KN199 was divided evenly into three parts, and these parts were treated in three different nutrient solutions containing different phosphorus concentrations (V0, V0.1, and V1). V1-treated KN199 showed the best growth, followed by V0.1-treated KN199, and V0-treated KN199 had the worst growth (Fig. 1A). On day 8, both V1 and V0.1-treated KN199 displayed the appearance of the first-stage tiller buds, whereas V0-treated wheat only had the development of first-stage axillary buds (Fig. 1B). From day 9, the difference in the development of three treatments was clearly observed. However, the difference between V1 and V0.1 was not as large as that observed between V0.1 and V0. From day 13, a significant difference was observed in the tillers of the three treatments; V1 and V0.1-treated wheat started to develop tillers, whereas V0 had no tillers. At day 20 as well, V0-treated KN199 had no tillers, and the tiller number was more in V1-treated KN199 compared with that in V0.1-treated KN199. Hence, we speculate that phosphorus deficiency hinders the wheat growth and plants would show less tiller in low phosphorus condition (Fig. 1C, D).

### Transcriptome results analysis

**Fig. 2:**
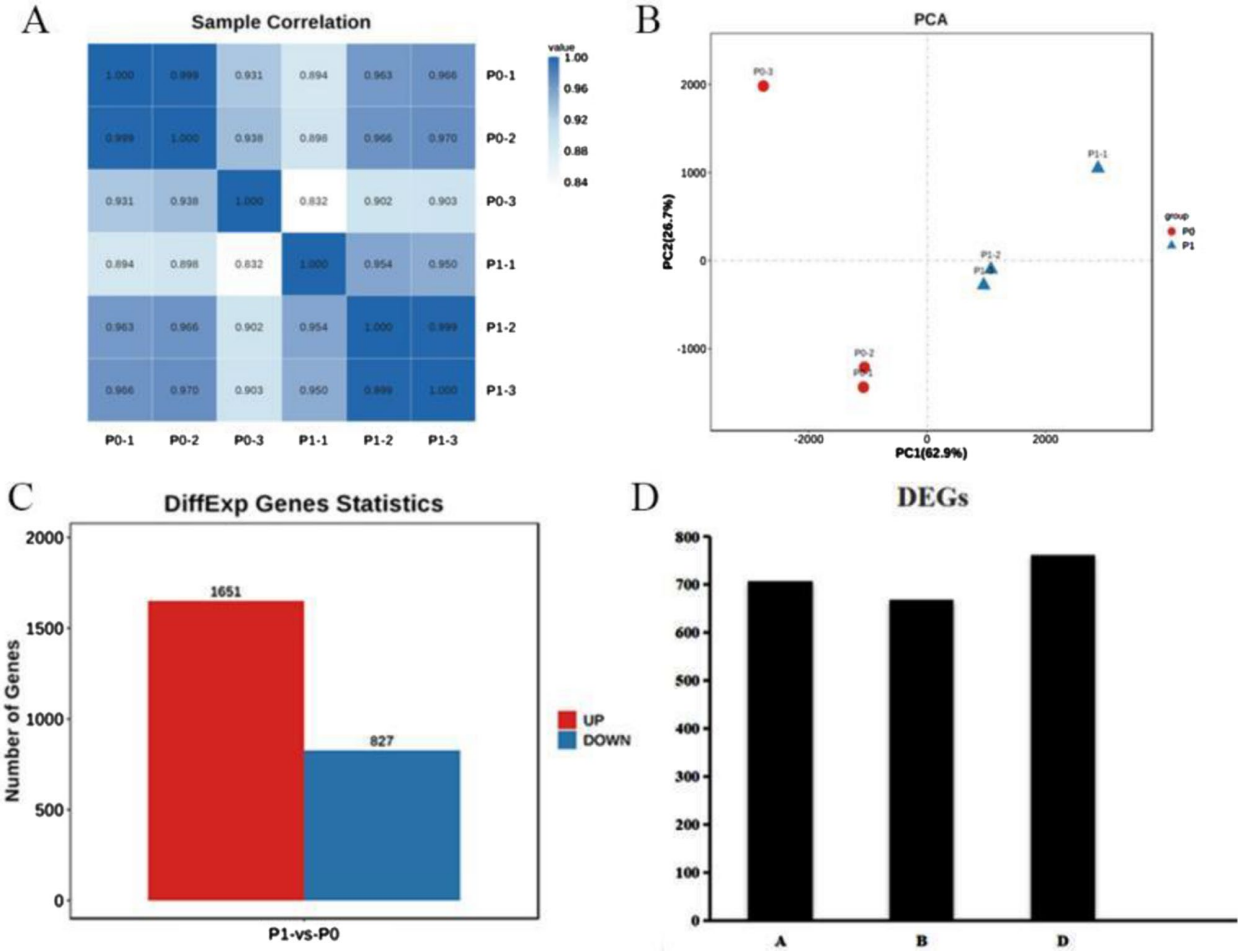
Quality analysis of RNA-seq transcriptome data. (**A**) Sample correlation heat map. (**B**) Principal Component Analysis. (**C**) Statistics of differentially expressed genes. (**D**) Total number of annotated genes in each genome

We performed correlation analysis of gene expression using the data of transcriptomics analysis and found acceptable reproducibility among samples (Fig. 2A), meeting the requirements for further analysis (Fig. 2B).

We identified a total of more than 120,000 wheat genes; of these, 2,478 were differentially expressed genes (DEGs) considering the screening criteria of FDR < 0.05 and |log2FC|>1. We found 1,651 upregulated and 827 downregulated genes (Fig. 2C) during the comparison of two sets of samples (Fig. 2D).

### GO and KEGG enrichment analysis of DEGs

**Fig. 3:**
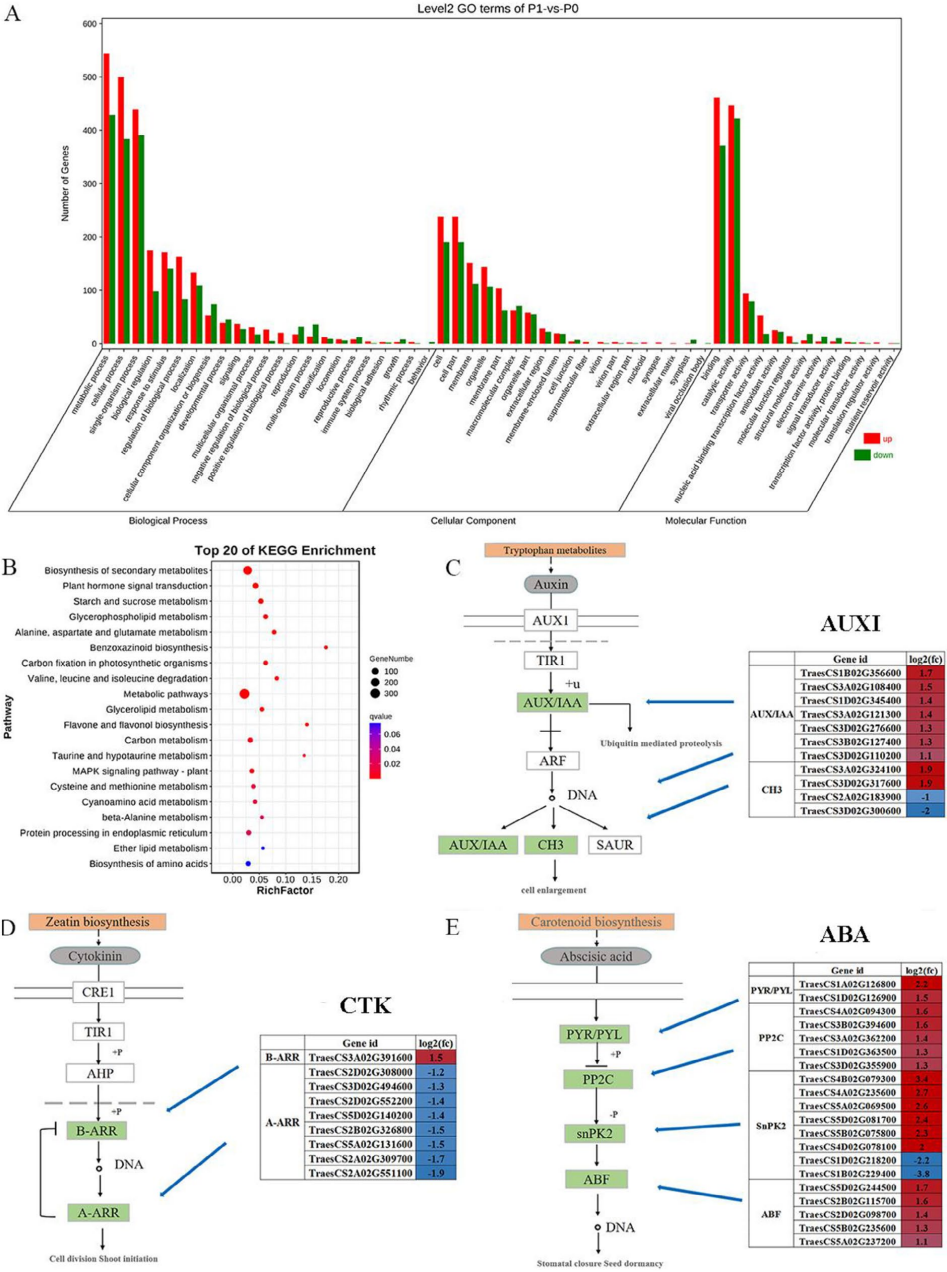
Differential gene clustering and major hormone signaling pathways. (**A**) Scatter plots of the GO pathway-enrichment analysis. (**B**) Scatter plots of the KEGG pathway enrichment analysis. (**C**) Genes with significant changes in expression levels in auxin signaling pathways. (**D**) Genes with significant changes in expression levels in cytokinin signaling pathways. (**E**) Genes with significant changes in expression levels in abscisic acid signaling pathways

Under low phosphorus stress, all the DEGs were classified into the following three major functional categories: Biological Process, Cellular Component, and Molecular Function. In all three categories, there were more upregulated genes than downregulated genes. In the Biological Process category, more DEGs were associated with pathways of metabolic process, cellular process, and single-organism process. In the Cellular Component category, most DEGs were present in cells. In the Molecular Function category, more DEGs were associated with molecular binding and catalytic reactions (Fig. 3A).

To perform functional classification and analyze the pathway enrichment of DEGs in wheat seedlings under low-phosphorus stress, the Kyoto encyclopedia of genes and genomes (KEGG) pathway enrichment analyses were carried out. DEGs were mainly enriched in metabolic pathways, biosynthetic pathways of secondary metabolites, and plant hormone signaling pathways (Fig. 3B). It is worth noting that the genes related to auxin, ABA, and cytokinin signal transduction were mainly enriched in plant hormone signaling pathways (Fig. 3C-E). It suggested that auxin, ABA, and cytokinin might participate in the regulation of axillary bud development by phosphorus treatment.

### Screening of the genes of interest

**Fig. 4:**
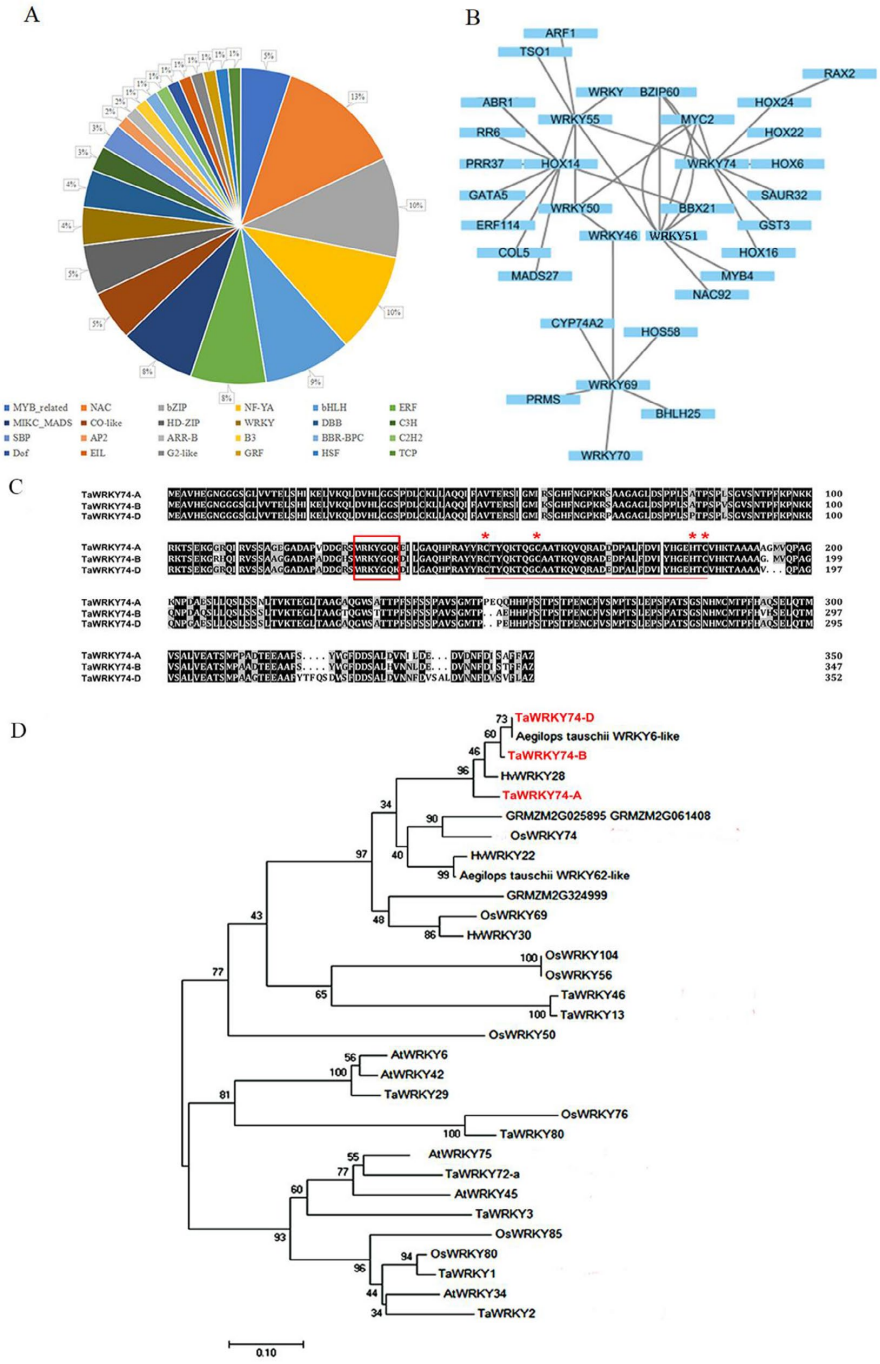
Acquisition of *TaWRKY74*s. (**A**) Classes of transcription factors in DEGs. (**B**) The interaction network of the regulation of tiller development in wheat. (**C**) Sequence alignment of TaWRKY74s (The conserved domain of WRKY family in the box). (**D**) Phylogenetic tree analysis of the TaWRKY74s

GO analysis suggested that TFs might play an important role in responding to low phosphorus stress. In this study, 43 putative TFs were identified. Of them, members of WRKY, MYB, NAC, bHLH, and ER family were abundant (Fig. 4A). The WRKY family is closely related to phosphorus stress, and hence we constructed a protein interaction map (Fig. 4B) with the WRKY TF family as the core by combining transcriptomics data STRING and predicted many genes related to low phosphorus stress and tiller development. Protein interaction network analysis showed that *TaWRKY74*s, *TaWRKY46*s, *TaWRKY6*9s, and other genes may be involved in the response of plants to the phosphorus stress. Since transcriptomic data suggested upregulation of *TaWRKY74-A*, *TaWRKY74-*B, *TaWRKY74-*D genes, we focused on studying the expression and role of *TaWRKY74*s.

### Isolation of *TaWRKY74* genes and its evolutionary tree analysis

*TaWRKY74*s have one copy each on A, B, and D chromosome, the total length of the genes is 1,050 bp, 1,041 bp, and 1,056 bp respectively. Those proteins had approximately 350 amino acid residues, with highly conserved WRKY family domain WRKYGQK (Fig. 4C). The evolutionary tree showed that TaWRKY74 proteins were closely related to OsWRKY74 of rice. The overexpression of OsWRKY74 significantly enhanced tolerance to Pi starvation. Under low phosphorus stresse,>24% increase in tiller number was observed in rice *OsWRKY74*-overexpressing plants compared with WT plants [18]. With *TaWRKY74* proteins have functional domains similar to OsWRKY74, we speculate that TaWRKY74s may have similar functions (Fig. 4D).

### Expression pattern of TaWRKY74 proteins

**Fig. 5:**
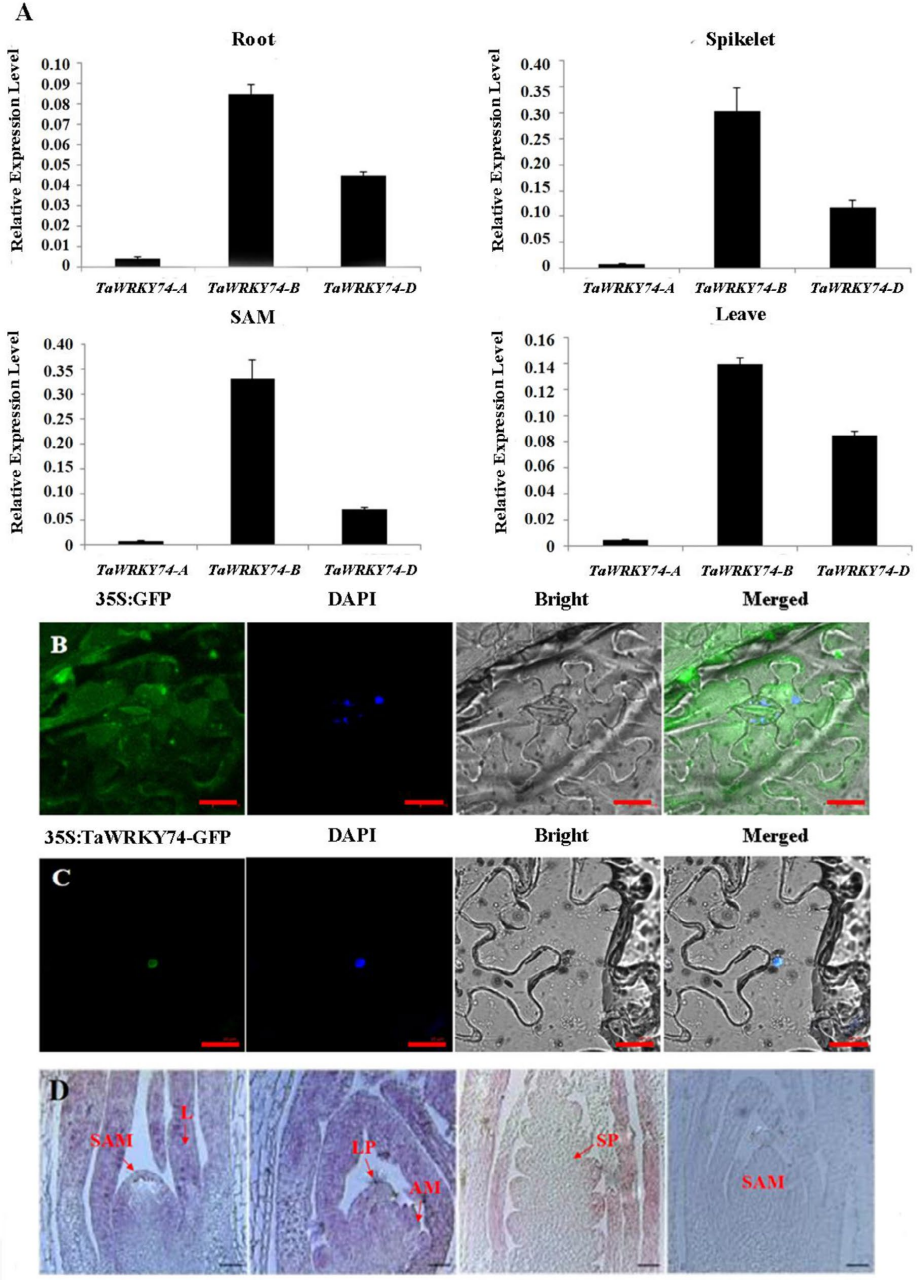
Acquisition of *TaWRKY74*s. (**A**) Quantitative analysis of TaWRKY74 proteins. (**B**) Subcellular localization of 35 S::GFP protein in tobacco leaves. (**C**) Subcellular localization of 35 S::TaWRKY74-B-GFP protein in tobacco leaves. Bar = 10 μm. (**D**) In situ hybridization analysis of *TaWRKY74*s, Bar = 100 μm. SAM: shoot apical meristem. AM: axillary meristem. L: leaf. SP: Spikelet primordium. LP:Leaf primordium

To study the expression pattern of TaWRKY74 proteins in SAM, leaves, spikelet primordia, young roots, and tillering nodes, we performed quantification, subcellular localization, as well as in situ hybridization experiments. We designed specific quantitative primers for *TaWRKY74-A*, *TaWRKY74-B*, and *TaWRKY74-D*, and determined their expression levels using cDNA isolated from the above plant tissue as templates. The results showed *TaWRKY74*s had a higher expression in SAM and spikelets, whereas the expression of *TaWRKY74-B* was higher in roots, spikelets, SAM, and leaves compared with those of *TaWRKY74-A* and *TaWRKY74-D* (Fig. 5A).

In the subcellular localization experiment which was performed with tobacco (*Nicotiana tabacum* L.), 35 S::TaWRKY74-B-GFP showed a strong signal only in the nucleus, indicating that TaWRKY74-B is localized in the nucleus (Fig. 5B-C). Given the high homology of TaWRKY74-A, TaWRKY74-B, and TaWRKY74-D proteins (> 92%) (Fig. 4C), we speculate that the other two proteins are both localized in the nucleus.

To further determine the expression of *TaWRKY74* genes in different tissues (axillary meristem, SAM, young leaves, leaf primordium, and spikelet primordium), the shoot tips in the signal ridge stage and the spikelets in the primordium stage were selected for in situ hybridization experiments, and the 350-650 bp region of *TaWRKY74*s was selected as a specific fragment synthesis probe. The result showed that TaWRKY74 proteins were all found to express in the above plant tissue (Fig. 5D).

### *TaWRKY74s*-RNAi plant phenotypic observation

**Fig. 6:**
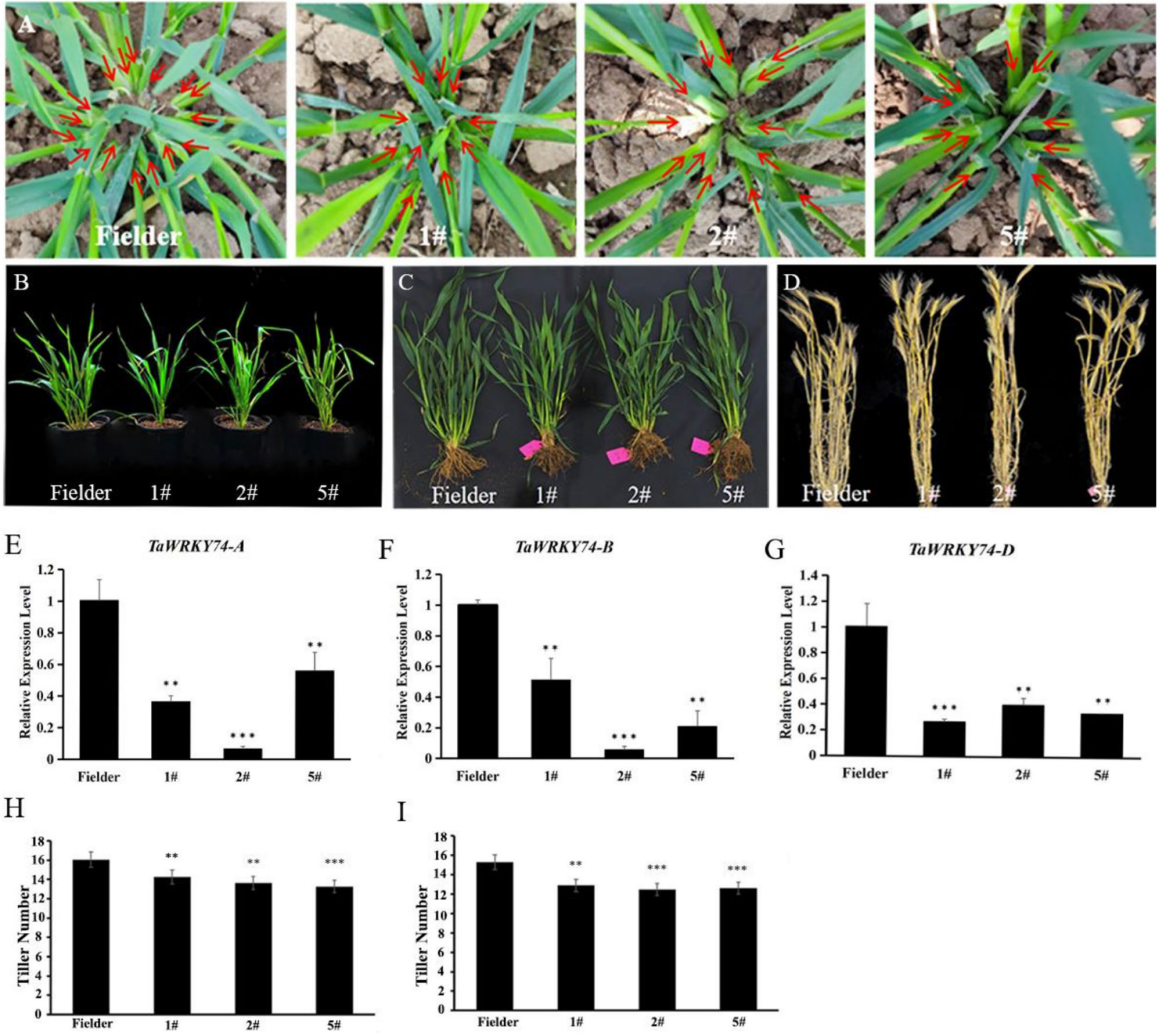
Phenotype observations of *TaWRKY74*s-RNAi plants in the field. (**A**) Tillers in wild-type Fielder and *TaWRKY74*s-RNAi plants at the jointing stage. (**B**) Phenotypic observations of plants subjected to low-phosphorus stress. (**C**) Wild-type Fielder and *TaWRKY74*s-RNAi plants at the jointing stage. (**D**) Wild-type Fielder and *TaWRKY74*s-RNAi plants at maturity. (**E**-**G**) Analysis of *TaWRKY74* expression in *TaWRKY74*s-RNAi transgenic plants. (Student’s t-test, *: p < 0.05, **:p < 0.01, ***: p < 0.001). (**H**) Tiller statistics at the jointing stage (n = 15, *: p < 0.05, **: p < 0.01, ***: p < 0.001). (**I**) Tiller statistics at the mature stage (n = 15, *: p < 0.05, **: p < 0.01, ***: p < 0.001)

Three transgenic lines, TaWRKY74s-RNAi-1#, TaWRKY74s-RNAi-2#, and TaWRKY74s-RNAi-5# were obtained and were treated with V0.1. In normal field environment, three liens of *TaWRKY74s*-RNAi transgenic wheat all showed reduced tiller number compare with wild-type (WT) plant; WT showed 15 to 17 tillers at the jointing stage while the tiller numbers in the RNAi lines reached 12 to 15 at the same stage (Fig. 6A and C). The effective tiller number of WT reached 14 to 16, whereas the effective tiller number of the RNAi lines reached 12 to 14 (Fig. 6D).

When WT and transgenic plants were treated under low phosphorus stress (V0.1), compared with WT, the tiller number reduction rate of transgenic strain was 26.7%, with a tiller number reduction rate of 14.6% under the field. These results indicated that TaWRKY74 proteins were related to phosphorus response and could affect the tiller number in wheat (Fig. 6B).

Compared with the wild type Fielder (WT), the expression levels of TaWRKY74-A, TaWRKY74-B, and TaWRKY74-D in the transgenic wheat were significantly downregulated (Fig. 6E-G). Therefore, we speculated that the reduced expression of TaWRKY74s affected the tiller number in wheat (Fig. 6H-I).

## Discussion

The uptake of phosphate (Pi) in plants from the soil is influenced by various factors. For instance, plants often enhance their efficiency of Pi absorption through modifications in growth and development processes or by interacting with microorganisms that facilitate Pi uptake [19]. Adaptive responses of plants to low Pi serve two primary functions: enhancing both the Pi acquisition and efficient use efficiently while protecting plants from the stress caused by low Pi. The reduction of wheat tiller under low Pi stress respresents a complex phenomenon involving multiple morphological and physiological alterations.

During the process of tiller development, there are significant changes in auxin levels. PIN protein is specific involved in to auxin transport. Alterations in the expression of *TaPIN1* could lead to the change in the tiller number in wheat [20]. *TaARF* plays an important role in regulating the auxin signaling pathway. *TaARF11* and *TaARF14* are important genes that participate in the regulation of wheat tillering through this pathway [21]. In miR167-overexpressing transgenic rice, the mRNA levels of *OsARF6*, *OsARF12*, *OsARF17*, and *OsARF25* were significantly decreased, resulting in a significant decrease in the tiller number [22]. Gene families, such as *AUX* and *IAA*, are also involved in auxin signaling pathways. For example, knocking out *OsAUX1* leads to a decreased auxin level and the tiller number. Furthermore, *OsIAA6*, which is an inhibitor of auxin signaling, also leads to a decrease in tillers [23]. Additionally, overexpression of IAA10 results in increased auxin levels and an increased tiller number [24]. Therefore,, it can be concluded that auxin is an important factor that affects the process of tiller development.

ABA plays an important role in the process of wheat resistance to low Pi stress. *BG* is the abscisic acid glucose ester decoupling gene; the expression of *BG1* and *BG2* was found to be upregulated under low Pi stress. In *Arabidopsis*, the ABA content in *aba2-1* and *bg1-1* mutants was lower compared to wild-type plants. While low Pi treatment induced an increase in ABA in wild-type plants, *aba2-1* mutants showed less extent of ABA increase while no ABA increase was observed in *bg1-1* mutants. This means that the release of ABA from ABA glucose esters by *BG1* plays an important role in the accumulation of ABA in plants induced by low Pi treatment [25]. PHT is a class of phosphorus transporters; *PHR1*, the positive regulator of PHT, is a core player in plant Pi starvation responses [26]. Plants often accurately regulate PHT at transcription and translation levels when responding to low phosphorus conditions *AtPHR1* activates the *AtPHT* expression by directly binding to the promoter of *AtPHT* to promote Pi absorption [26]. In our study, the KEGG analysis showed that a few genes, such as *ARF*(MSTRG.40,419), *BG(Traescs3D02G493600LC)*, and *PHT(TraesCS5B02G470100)*, related to auxin and ABA metabolism in wheat under low phosphorus stress displayed significant changes in expression. We speculate that auxin and ABA pathway in wheat changes under the low Pi stress.

**Fig. 7:**
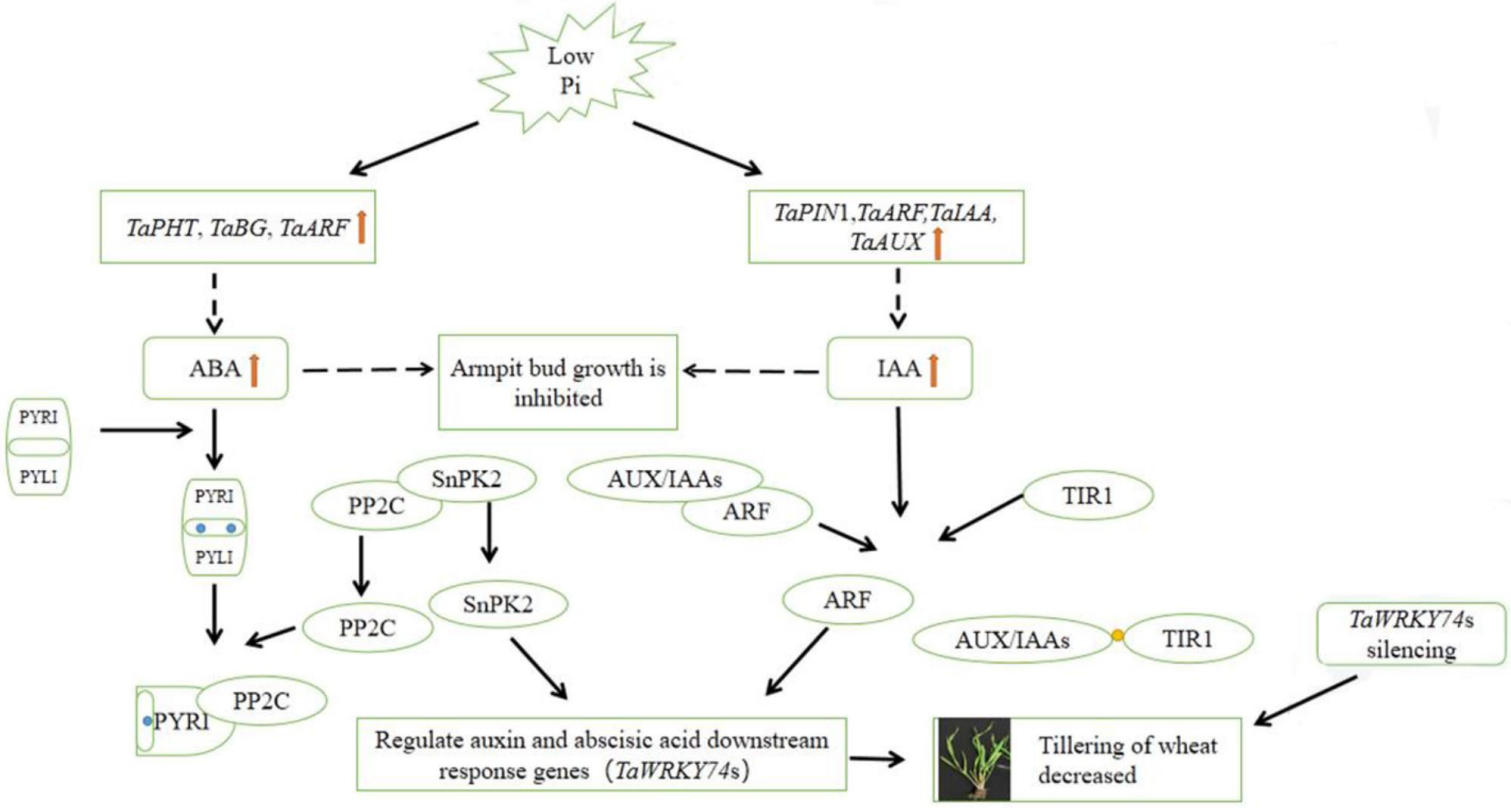
Molecular model of low phosphorus stress regulating tillering in wheat

In this study, *TaWRKY74* gene was identified as a crucial factor in influencing wheat tillering under low Pi stress. When we analyzed the promoter structure of this gene, a few auxin and ABA-binding elements were found, including TGTCTC [27]. These finding and the KEGG pathway results suggest that *TaWRKY74* may affect wheat tillering through auxin and ABA pathways. We have predicted a molecular model of regulating tillering in wheat under low phosphorus stress (Fig. 7). The expression of many genes and the content of auxin and ABA changed in wheat under the low Pi stress. We hypothesize that these changes have two effects on wheat tillering. First, the changes in hormone concentration directly affect the growth and development of axillary buds [28–30], and second, adjusting the content of SnPK2 and ARF could lead to the binding to the downstream target genes, thereby, affecting tiller developmentt via changed hormone concentration [31].

## Conclusions

The results of this study indicate that *TaWRKY74*s are related to phosphorus reactions and may affect the number of tillers in wheat. This research provides potential directions and valuable resources for cultivating low phosphorus tolerant wheat varieties in the further.

## Materials and methods

### Plant materials and stress treatments

Three Hoagland nutritional solutions were set up, each with a distinct quantity of phosphorus: 0 mmol/L(V0), 0.1 mmol/L(V0.1), and 1 mmol/L (V1).

We started by vernalizing the wheat cultivar KN199 for 30 days (18 h of light and 6 h of darkness, with an ambient temperature of 4 °C). We next chose wheat that grew consistently and treated it with varying amounts of phosphorus (V0, V0.1, and V1) at 16 h of light and 8 h of darkness, with the temperature of 24℃ for 7 days, monitoring its growth condition every day. We then re-vernalized a batch of the wheat cultivar KN199 seeds and selected tillering site of the wheat, which was transferred to the greenhouse on the eighth day. The wheat material treated with V1 was named P1, and the wheat material treated with V0.1 was named P0. RNA was extracted from the two materials and sent to the transcriptome for sequencing. Experiments were conducted in three biological replicates per set of samples.

### Screening and acquisition of TaWRKY74 *protein*

Transcriptome data was used to screen out all transcription factors and build a protein interaction network based on STRING (https://cn.string-db.org/) and Cytoscape software. We selected a few significantly expressed genes; *TaWRKY74* was identified among these genes.

### Cloning and evolutionary tree analysis of *TaWRKY74*s

Using specifically-designed primers(Table S1), PCR amplification was conducted using cDNA isolated from the stem of the wheat KN199.

The protein sequences of TaWRKY74s were retrieved and analyzed in NCBI (https://www.ncbi.nlm.nih.gov/) using BLAST. The DNA sequences accession of *TaWRKY74*-A is CM022223, protein accession is KAF7058608. The DNA sequences accession of *TaWRKY74*-B is CM022221, protein accession is KAF7049107. The DNA sequences accession of *TaWRKY74*-D is CM022225, protein accession is KAF7072215. To construct an evolutionary tree, highly homologous protein sequences from different species were aligned, and the tree was prepared using MEGA7 software.

### Protein sequence and promoter analysis of *TaWRKY74*s

The amino acid sequences of TaWRKY74-A, TaWRKY74-B, and TaWRKY74-D were compared using DNAClub and DNAMAN softwares. WheatOmics 1.0 (http://202.194.139.32/) was used to find the first 4000 bp sequence of *TaWRKY74*s promoter, and the database GSDS2.0 (http://gsds.gao-lab.org/index.php) was used for the promoter analysis.

### The construction and selection of transgenic RNAi wheat plants

For the RNA-interfering plasmid construct, cDNA was amplified by PCR using the primers *TaWRKY74*-RNAi-F and *TaWRKY74*-RNAi-R (Supplementary material: Table S1). The PCR product was inserted into the RNAi vector PC336 using the Gateway entry vector system to trigger specific RNAi of *TaWRKY74*s in wheat, which created a construct, namely, *TaWRKY74*s-RNAi. Then, the construct was transformed into the wheat cultivar Fielder. After obtaining the transformed seedlings, three *TaWRKY74*s-RNAi-positive plants were obtained by polymerase chain reaction (PCR)-based identification. Bar (selective marker gene) and *TaWRKY74*s genes were confirmed simultaneously in transgenic positive lines.

### Quantitative real-time PCR

The SAM, leaves, spikelet primordium, young roots of WT wheat, and the tillering joints of TaWRKY74s-RNAi strain exposed to low phosphorus stress were used to extract the total RNA using Trizol reagent (Invitrogen). Total RNA (2 µg) was reverse-transcribed into cDNA using reverse transcription kits. Each sample (1 µg cDNA) was used to perform the first RT-PCR using SYBR Green Master Mix. The Cq value was used to dilute cDNA of all samples to the same concentration. Subsequently, a second RT-PCR and data analysis were conducted.

### Localization of *TaWRKY74s-*GFP fusion proteins

The full-length cDNA of *TaWRKY74-B* was ligated into vector *35 S::GFP* to construct fusion protein expression vector *35 S::TaWRKY74-B-GFP*. *Agrobacterium* tobacco injection method was used to transiently transfect the bacterial solution with the target fragment plasmid, and the control only 35 S::GFP was injected into the epidermis of tobacco. After 36 h, the epidermis of tobacco leaves was teared using forceps and the GFP was observed using the confocal microscope.

### In situ hybridization

The 350-650 bp region on TaWRKY74s was selected as a specific fragment synthesis probe. WT wheat was used as the material, and the stem tips of wheat in the monoprism stage and the wheat spikelets in the primordium stage of the spikelets were selected for in situ hybridization experiments. The wheat stem apex was fixed in 4% v/v paraformaldehyde and 0.1 M phosphate buffer (pH 7.0) overnight at 4℃. Then, the specimens were embedded in paraplast (Sigma, USA) and sectioned at 8.0 μm. Antisense and sense RNA probes were synthesized using a digoxigenin RNA labeling kit (Sigma-Aldrich, USA). The RNA probe for *TaWRKY74*s genes was amplified by PCR using the primers in Table S1 of supplementary material. The PCR procedure was performed according to the instructions for the 2×Phanta Max Master Mix from Vazyme. The hybridization and the detection of hybridized signals were carried out as described by Wang et al. [32].

### Transcriptome sequencing and data analysis

Total RNA was extracted using the RNA simple Total RNA kit (Tiangen Biotech Co., Ltd). The products were sequenced using an Illumina Novaseq 6000 (Gene Denovo Biotechnology Co.). RNA-seq was performed using three biological replicates and the data have been deposited in the Genome Sequence Archive [33] in the National Genomics Data Center [34], China National Center for Bioinformation/Beijing Institute of Genomics, Chinese Academy of Sciences (GSA: CRA012272). These data are publicly accessible at https://bigd.big.ac.cn/gsa/browse/CRA012272. The filtered clean reads were mapped to the wheat reference genome and genes (http://plants.ensembl.org/Triticum_aestivum/lnfo/lndex).

The mapped reads from each sample were assembled using StringTie v1.3 in a reference-based approach [35]. For each transcription region, a fragment per kilobase of transcript per million mapped reads (FPKM) value was calculated to quantify the level of expression and variations using RSEM software. KEGG is a major database used for the assessment of gene enrichment in biological pathways [36]. Pathway enrichment analysis showed that the DEGs were significantly enriched in pathways associated with metabolism and signal transduction, in comparison with the overall genomic background [37]. The calculated p-values were FDR corrected, using FDR ≤ 0.05 as the threshold. Pathways meeting this condition were defined as significantly enriched in DEGs. Subsequently, the KEGG pathway and GO analyses were conducted (Fig. 3A and B).

### Electronic supplementary material

Below is the link to the electronic supplementary material.

Supplementary Material 1: Table S1 All the specific primers used in this research.

## Data Availability

In this study, we used the wheat cultivar KN199 and *Nicotiana benthamiana*. Both of them are publicly available breeds,we have purchased them. The RNA-seq data were deposited in the Genome Sequence Archive in National Genomics Data Center, China National Center for Bioinformation/Beijing Institute of Genomics, Chinese Academy of Sciences (GSA:CRA012272) that are publicly accessible at https://bigd.big.ac.cn/gsa/browse/CRA012272. All the original data and materials of this study are also kept in our laboratory. Please contact us at any time if necessary. Email: wangf@sdau.edu.cn;tangheng2018@sdau.edu.cn.
