# Supplementary material for: Transcriptome analysis of axillary buds in low phosphorus stress and functional analysis of TaWRKY74s in wheat
Source: BMC Plant Biol. 2024 Jan 2;24:1. doi: 10.1186/s12870-023-04695-w (PMC10759677; doi:10.1186/s12870-023-04695-w)
Supplement: Supplementary file 1 — Supplementary Material 1: Table S1 All the specific primers used in this research. [file 12870_2023_4695_MOESM1_ESM.docx]

Supplementary material:

**Table S1** All the specific primers used in this research.

| Primers | Primers [Sequence](D:/Dict/8.5.1.0/resultui/html/index.html#/javascript:;)(5’-3’) | [Purpose](D:/Dict/8.5.1.0/resultui/html/index.html#/javascript:;) | Annealing Temperature(℃) |
| --- | --- | --- | --- |
| *RTQWRKY74-A-*F | CCATGCCAACAACAAGAAGAG | Quantitative real-time PCR | 56 |
| *RTQWRKY74-A-*R | ACACATACACCCACACTAACC | Quantitative real-time PCR | 55 |
| *RTQWRKY74-B-*F | GCCTTTCCATACCAACAACAAG | Quantitative real-time PCR | 56 |
| *RTQWRKY74-B-*R | TGAGCGGGTGATACCTCTAATA | Quantitative real-time PCR | 56 |
| *RTQWRKY74-D-*F | CACACCTGCGTCCATAAGA | Quantitative real-time PCR | 56 |
| *RTQWRKY74-D-*R | CTCAGGCAAGGAAGCTAGAA | Quantitative real-time PCR | 55 |
| *WRKY74-A-*RNAI-F | CCATGCCAACAACAAGAAGAG | Quantitative real-time PCR | 56 |
| *WRKY74-A-*RNAI-R | ACACATACACCCACACTAACC | Quantitative real-time PCR | 55 |
| *WRKY74-B-*RNAI-F | GCCTTTCCATACCAACAACAAG | Quantitative real-time PCR | 56 |
| *WRKY74-B-*RNAI-R | TGAGCGGGTGATACCTCTAATA | Quantitative real-time PCR | 56 |
| *WRKY74-D-*RNAI-F | CACACCTGCGTCCATAAGA | Quantitative real-time PCR | 56 |
| *WRKY74-D-*RNAI-R | CTCAGGCAAGGAAGCTAGAA | Quantitative real-time PCR | 55 |
| *Actin*‑F | AGTCGAGAACGATACCAGTAGTACGA | Quantitative real-time PCR | 59 |
| *Actin*‑R | GCCATGTACGTCGCAATTCA | Quantitative real-time PCR | 57 |
| *TaWRKY74*-F | CCGAGCAGAGAGAGATGGAG | Gene amplification | 57 |
| *TaWRKY74*-R | CTGCACTGAGGAAGAACTGGCGTG | Gene amplification | 63 |
| *TaWRKY74*-RNAi-F | CACCATGGAGGCCGTGCATGAGG | Vector construction | 66 |
| *TaWRKY74*-RNAi-R | TGCGAAGAAGGCAGAAATATCG | Vector construction | 57 |
| *Sense probe*-F | GAATTGTAATACGACTCACTATAGGGACGGAGCTGAGCCACATCAA | In situ hybridization | 68 |
| *Sense probe*-R | CTGGTACGTGCAACGGTAGTATG | In situ hybridization | 59 |
| *Antisense probe*-F | ACGGAGCTGAGCCACATCAA | In situ hybridization | 60 |
| *Antisense probe*-R | GAATTGTAATACGACTCACTATAGGGCTGGTACGTGCAACGGTAGTATG | In situ hybridization | 68 |
